# Minimally Invasive Treatment of Three-Part Proximal Humerus Fractures: A Two-Center Comparative Study of Plate Fixation and Intramedullary Nailing

**DOI:** 10.3390/jcm14217880

**Published:** 2025-11-06

**Authors:** Calogero Puma Pagliarello, Vito Pavone, Fabrizio Quattrini, Pietro Maniscalco, Virginia Masoni, Corrado Ciatti

**Affiliations:** 1 Department of Orthopedics and Traumatology, Guglielmo da Saliceto Hospital, Via Taverna 49, 29121 Piacenza, Italy; c.pumapagliarello@ausl.pc.it (C.P.P.); fabrizioquattrini@yahoo.it (F.Q.); 2 Department of General Surgery and Medical-Surgical Specialties, A.O.U. Policlinico Rodolico-San Marco, University of Catania, Via Santa Sofia, 78, 95123 Catania, Italy; vitopavone@hotmail.com; 3 Department of Medicine and Surgery, University of Parma, 43126 Parma, Italy; 4 Department of Medicine and Aging Sciences, University of Chieti-Pescara, 66100 Chieti, Italy; pietro.maniscalco@unipr.it; 5 Department of Orthopedics and Traumatology, University of Turin, Via Zuretti, 29, 10126 Turin, Italy

**Keywords:** proximal humerus fracture, plate osteosynthesis, intramedullary nailing, minimally invasive surgery

## Abstract

**Background/Objectives:** Proximal humerus fractures account for approximately 5% of all skeletal injuries, and their optimal surgical management remains debated. The optimal fixation method for three-part proximal humerus fractures remains a matter of debate. This study aimed to compare the clinical and radiological outcomes of two minimally invasive osteosynthesis techniques—plate fixation and intramedullary nailing—for the treatment of three-part proximal humerus fractures. **Methods:** Sixty-six patients aged 60–80 years were retrospectively analyzed across two centers adopting different institutional preferences. Thirty-three patients were treated with minimally invasive plate fixation and thirty-three with intramedullary nailing. The mean age was 67.8 ± 4.2 years, and the mean follow-up duration was 27.2 months. Functional and clinical outcomes were evaluated using the Barthel Index, DASH, Simple Shoulder Test (SST), and Visual Analog Scale (VAS). Operative time, hospitalization length, healing time, and postoperative complications were recorded and statistically analyzed. **Results:** Intramedullary nailing was associated with shorter operative and hospitalization times and fewer complications. Early SST and VAS improvements favored the nailing group, while long-term outcomes were comparable. **Conclusions:** Intramedullary nailing represents a reliable and less invasive alternative to plate fixation in the treatment of three-part proximal humerus fractures. It offers shorter operative and hospitalization times, fewer postoperative complications, and faster functional recovery while achieving comparable long-term outcomes. Surgeon experience and familiarity with the chosen technique remain key determinants of success.

## 1. Introduction

Fractures of the proximal humeral epiphysis represent approximately 5% of all fractures, increasing to 10% among elderly patients, a significant public health concern given the aging population [1]. Domestic environments are the most common setting for such injuries, accounting for 50% of cases in the general population and up to 90% among individuals over 60 years old. In younger patients, these fractures typically result from high-energy trauma such as falls from height, road traffic accidents, or sports injuries [2].

The first-line treatment for proximal humeral fractures is conservative management, aimed at restoring a painless range of motion. This approach is generally reserved for simple or minimally displaced fractures or for patients with low functional demands and multiple comorbidities [3]. Approximately 20% of proximal humerus fractures require surgical intervention [4].

When surgery is indicated, several options are available, including open reduction and internal fixation (ORIF) with plates, closed reduction and intramedullary nailing, percutaneous fixation with K-wires, and shoulder arthroplasty [4,5,6,7,8]. Each method presents advantages and disadvantages related to multiple factors:

Patient-related factors include age, pre-injury autonomy, substance abuse (which increases the risk of nonunion, wound dehiscence, and poor compliance), comorbidities (predisposing to infection or osteonecrosis), and osteoporosis (which compromises fixation stability and bone healing). Functional demand and life expectancy also influence treatment decisions [9,10,11,12]. Trauma-related factors include fracture type, degree of displacement, soft-tissue conditions, and associated injuries. Surgeon-related factors involve technical experience and the availability of fixation devices.

Radiographic evaluation remains the diagnostic gold standard, typically involving anteroposterior and axillary views [13,14,15,16,17]. In complex cases where the treatment choice is uncertain, CT scanning is recommended. Accurate fracture classification is essential for management planning, providing prognostic insights and guiding treatment selection. The most widely used classification systems are those of Neer [18], the AO/OTA (Arbeitsgemeinschaft für Osteosynthesefragen/Orthopedic Trauma Association) [19], and Hertel [20].

K-wire fixation preserves soft-tissue integrity but often provides insufficient stability for early mobilization; thus, it is generally replaced by open reduction. Plate fixation is among the most commonly performed procedures for three-part fractures [21,22]. The most frequently employed surgical approaches are the deltopectoral approach, which ensures good exposure while preserving the axillary nerve, and the transdeltoid approach, which facilitates reduction and fixation [23]. Alternatively, intramedullary nailing, especially for metaphyseal fractures, can be performed via a transdeltoid approach. The rotator cuff should be incised along the fiber direction to preserve muscle integrity. Nailing is considered more conservative with respect to soft tissues and is associated with reduced blood loss, a lower risk of neurovascular injury, fewer adhesions, and a reduced infection rate, though it may carry a higher risk of avascular necrosis and potential rotator cuff injury during removal [24,25].

Clinical and functional outcomes reported in the literature for three-part fractures treated with plates or nails are comparable, both proving superior to conservative treatment [26]. However, the choice of fixation technique also depends on the surgeon’s expertise and device familiarity.

Despite several comparative studies, the optimal fixation method for displaced three-part fractures in elderly patients remains controversial. Most available data derive from heterogeneous populations or single-center experiences, and few studies have directly compared modern minimally invasive techniques under standardized inclusion criteria. This gap justifies the present investigation, which aims to clarify the relative clinical efficiency of plate fixation and intramedullary nailing in comparable elderly cohorts.

The purpose of this study was to compare plate fixation and intramedullary nailing for three-part proximal humerus fractures in patients over 60 years old, focusing on clinical, radiological, and functional outcomes as well as complications.

## 2. Materials and Methods

A retrospective observational study was conducted by analyzing clinical and radiological data collected from two Italian orthopedic centers: the Orthopedic Clinic of Catania (Sicily, Southern Italy) and the Guglielmo da Saliceto Hospital in Piacenza (Emilia-Romagna, Northern Italy). The research design was based on the distinct institutional protocols used in the two facilities. In Catania, mini-invasive plating with screws represents the standard of care for three-part proximal humerus fractures, whereas in Piacenza, intramedullary nailing is the preferred method. This natural differentiation provided an ideal framework for a comparative investigation between the two surgical techniques.

Between January 2020 and December 2021, 115 consecutive patients with proximal humerus fractures were screened for eligibility. Inclusion criteria were strictly defined as follows: age between 60 and 80 years, absence of significant systemic comorbidities (e.g., advanced cardiopulmonary disease, severe diabetes), no previous upper limb functional deficit, and diagnosis of a three-part proximal humerus fracture according to Hertel’s classification, indication for surgical treatment, and patient’s acceptance of the procedure [27]. The age range of 60–80 years was chosen to include patients with adequate bone quality for fixation while excluding very frail or non-ambulatory individuals. Fracture classification according to Hertel was performed independently by two orthopedic surgeons, with disagreements resolved by consensus (κ = 0.86).

All patients had been treated using either minimally invasive plate fixation or intramedullary nailing. To ensure homogeneity, only specific implant systems were included: the NCB^®^ Proximal Humerus System (Zimmer Inc., Warsaw, IN, USA) for the plate group and the Diphos^®^ short nail (Lima Corporate, San Daniele del Friuli, Italy) for the nail group.

Exclusion criteria comprised fracture–dislocations, head-splitting fractures, pathological or open fractures, associated severe ipsilateral shoulder injuries, and any neurological or vascular involvement of the affected limb. Patients who died or were lost to follow-up were also excluded from the final statistical analysis.

**Figure 1:**
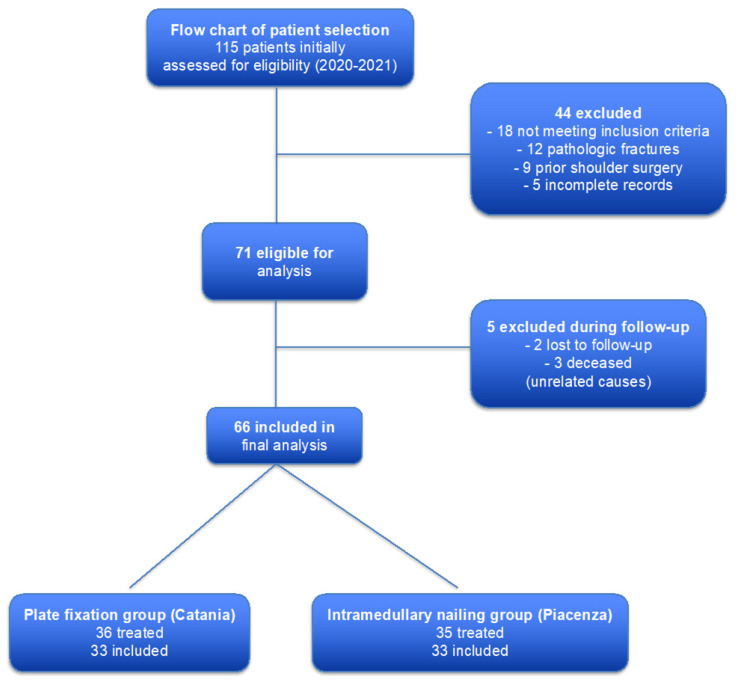
Patient screening and enrollment. The main pathway shows the count of patients screened, eligible, and included; exclusion branches are displayed on the right-hand side and visually separated from the main flow. Of 115 patients screened, 71 met eligibility criteria; after losses to follow-up and unrelated deaths, 66 were analyzed (33 plating, 33 nailing).

In Catania, 36 patients were treated with minimally invasive plating, whereas in Piacenza, 35 patients underwent intramedullary nailing. A total of 71 patients (61.7% of the total) were initially enrolled. Fracture classification according to Hertel revealed 27 type 7 patterns (38.0%), 18 type 8 (25.3%), 15 type 9 (21.1%), 7 type 10 (8.8%), and 4 type 11 (5.6%). Among these, 35 patients (49.3%) underwent intramedullary nailing and 36 (50.7%) plate fixation. Five patients were excluded during the observation period due to loss to follow-up (two cases) or death from unrelated causes (three cases). The final sample therefore consisted of 66 patients (mean age 67.8 ± 4.19 years), evenly distributed between the two treatment groups (*n* = 33 per group) (Figure 1).

All procedures were performed by experienced orthopedic surgeons specialized in upper limb trauma surgery. Preoperative assessment included clinical examination and radiographic evaluation with standard anteroposterior and axillary views. In selected complex cases, CT scans were used to improve surgical planning and to confirm fracture classification. The operative technique was performed under general anesthesia or interscalene block. In the plate group, all procedures were carried out through a minimally invasive transdeltoid approach, which provided direct lateral exposure of the proximal humerus and allowed for accurate reduction and plate positioning under fluoroscopic control. In the nail group, fixation was achieved through a small (approximately 2 cm) anterolateral skin incision, followed by a limited incision of the supraspinatus tendon along the direction of its fibers to reach the nail entry point. The rotator cuff was systematically repaired at the end of the procedure using nonabsorbable sutures. Both surgical techniques aimed to achieve anatomical reduction and stable fixation, permitting early postoperative mobilization and functional rehabilitation.

Postoperatively, all patients followed the same rehabilitation protocol, beginning with pendulum exercises within the first postoperative week, progressing to passive-assisted range of motion after two weeks, and to active exercises at four to six weeks, depending on pain and radiological findings. Full activity was generally resumed after radiographic evidence of union.

Clinical and functional evaluations were conducted at 1, 3, 6, and 12 months postoperatively and at a final follow-up (mean duration 27.2 months). Functional outcomes were assessed using the Disabilities of the Arm, Shoulder, and Hand (DASH) score [28], the Barthel Index for activities of daily living [29], the Simple Shoulder Test (SST) [30], and the Visual Analog Scale (VAS) for pain [31]. Functional evaluations were performed by independent orthopedic specialists not involved in the surgery, although complete blinding to the treatment type was not feasible. Radiographic assessments included monitoring for secondary displacement, implant mobilization, delayed union, nonunion, avascular necrosis of the humeral head, screw cut-out, and post-traumatic arthritis. Avascular necrosis (AVN) was diagnosed based on serial radiographs showing late segmental collapse of the humeral head, sclerosis, or subchondral lucency (Cruz classification stages II–IV). MRI was used only when radiographic findings were inconclusive. Statistical analysis was performed using standard parametric and nonparametric tests, with significance set at *p* < 0.05.

### 2.1. Statistical Analysis

All data collected from both centers were entered into a dedicated electronic database and analyzed using IBM SPSS Statistics version 27.0 (IBM Corp., Armonk, NY, USA). Continuous variables were expressed as mean ± standard deviation (SD) and range, while categorical variables were expressed as absolute frequencies and percentages. The Shapiro–Wilk test was applied to assess data normality. For continuous variables, Student’s *t*-test for independent samples was used when data were normally distributed, while the Mann–Whitney U test was employed for nonparametric distributions. Categorical variables were compared using the chi-square test or Fisher’s exact test, as appropriate. Repeated measures analysis of variance (ANOVA) was used to compare functional scores at different time points (1, 3, 6, and 12 months). A linear mixed-effects model was applied with random patient intercept and fixed effects for treatment, time, and treatment × time interaction. Covariates included age, sex, Hertel subtype, and center. Adjusted mean differences (95% CI) were reported.

A *p*-value < 0.05 was considered statistically significant. Effect sizes were calculated to assess the clinical relevance of differences, and 95% confidence intervals (CIs) were reported where applicable. Missing data were handled using pairwise deletion to retain maximal statistical power without introducing bias.

In addition to univariate comparisons, functional outcomes (SST, DASH, VAS, Barthel Index) were analyzed using a linear mixed-effects model with random intercepts for subjects and fixed effects for treatment, time, and treatment × time interaction. Age, sex, Hertel subtype, and center were included as covariates. Adjusted mean differences with 95% confidence intervals were reported.

Missing data (<10%) were handled within the mixed-effects framework under the assumption of missing at random, thus avoiding pairwise deletion.

A post hoc power analysis was performed on the main functional outcome. Based on an effect size (Cohen’s d) of 0.65, α = 0.05, and the actual sample size (*n* = 33 per group), the achieved power was 0.83, confirming that the study was sufficiently powered to detect clinically meaningful differences.

### 2.2. Use of GenAI in Writing

Artificial Intelligence-based tools (ChatGPT, OpenAI, USA) were used exclusively for English language editing and grammar refinement. The authors reviewed and approved all generated text to ensure accuracy and full responsibility for the final manuscript content.

## 3. Results

A total of 66 patients completed the study protocol and follow-up assessments. Demographic characteristics were comparable between groups, with no statistically significant differences in mean age, sex distribution, dominant side involvement, or fracture type distribution according to Hertel’s classification (Table 1). The mean follow-up duration was 27.2 months (range 18–36 months), providing adequate time to assess both functional recovery and long-term complications.

**Figure 2:**
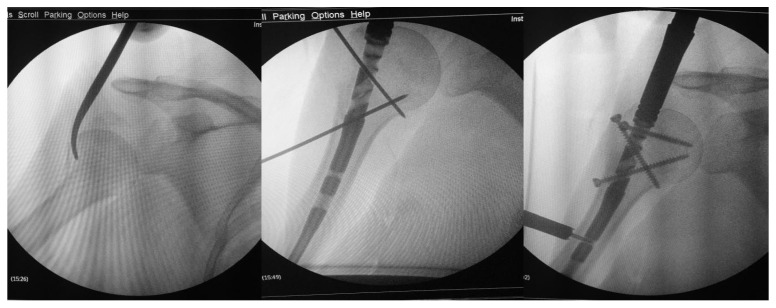
Intraoperative images. Surgery was performed through a percutaneous reduction and a minimally invasive nailing.

Operative time, hospitalization period, and bone healing duration were significantly shorter in the nailing group. The mean operative time for plate fixation was 61 min, compared to 27 min for nailing (−55.7%, *p* < 0.05) (Figure 2). Hospitalization averaged 9.2 days for patients treated with plates and 6.3 days for those with nails (−31.5%, *p* < 0.05). The mean time to radiographic healing was 87.5 days for plates and 78.4 days for nails (−10.4%, *p* < 0.05). Preoperative waiting time did not differ between groups and matched Table 1 values (2.3 ± 0.9 vs. 2.1 ± 1.0 days; *p* = 0.44). Operative time was significantly shorter for nailing (27.3 ± 5.9 min) than plating (62.1 ± 9.7 min); mean difference −34.8 [95% CI, −38.2 to −31.4], *p* < 0.001. Hospital stay averaged 6.3 ± 2.1 vs. 9.2 ± 2.5 days (mean difference −2.9 [95% CI, −3.7 to −2.1], *p* < 0.001). Time to union was 78.4 ± 14.7 vs. 88.2 ± 18.3 days (*p* = 0.04). Early SST and VAS differences remained significant at one month (*p* < 0.01).

Radiographic assessment included measurement of head–shaft angle, GT position, calcar buttress integrity, and screw tip–cortex distance. Restoration of the neck–shaft angle averaged 133° ± 7° for nailing and 131° ± 8° for plating (*p* = 0.41). No significant correlation was found between these metrics and final functional scores, but loss of reduction and avascular necrosis were associated with initial malreduction (*p* < 0.05).

Functional outcomes improved progressively throughout follow-up in both groups. At final evaluation, the Barthel Index averaged 84.1 for the plate group and 80.4 for the nail group. DASH scores were 37.3 (plate) versus 40.5 (nail), indicating a comparable degree of upper limb functionality. SST scores were slightly higher in the nail group (63.8 vs. 56.8), and VAS pain levels were lower (2.3 vs. 3.27). While these differences were not statistically significant at final follow-up, the SST and VAS scores demonstrated a significant advantage for the nailing group at one month post-surgery (*p* < 0.05), suggesting a faster initial recovery (Table 2).

A total of 26 complications were recorded: 16 in the plate group and 10 in the nail group, corresponding to overall complication rates of 48.5% and 30.3%, respectively (Table 3). The most frequent complications in the plate group were humeral head necrosis (4 cases), loss of reduction (3), delayed union (3), nonunion (1), and malunion (4). In the nail group, complications included screw cut-out (2 cases), intraoperative fractures (1), rotator cuff lesions (1), delayed union (1), and malunion (4). No cases of deep infection were recorded in either group. The distribution of complications according to Hertel’s classification showed a tendency toward higher complication rates in more complex fracture types (types 9–11).

**Figure 3:**
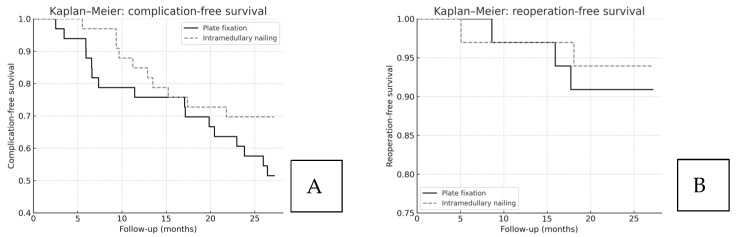
Kaplan–Meier analysis for (**A**) complication-free survival and (**B**) reoperation-free survival. Solid line: plate fixation; dashed line: intramedullary nailing. Legend positions and censoring markers are standardized across panels. No significant differences were observed (log-rank *p* = 0.29 and *p* = 0.47, respectively).

Kaplan–Meier analysis showed no significant difference in overall complication-free survival between plate fixation and intramedullary nailing (log-rank *p* = 0.29). The estimated hazard ratio for complications (plate vs. nail), adjusted for center and Hertel subtype, was 1.31 (95% CI 0.69–2.48). Reoperation-free survival was likewise comparable between groups (log-rank *p* = 0.47). These data are illustrated in Figure 3.

Reoperations were required in five patients: three in the plate group (9.1%) and two in the nail group (6.1%). In the plate group, two reoperations were performed for humeral head necrosis, both requiring prosthetic replacement, and one for nonunion treated by revision plating. In the nail group, reoperations involved screw repositioning due to cut-out and correction of minor loss of reduction. The lower rate and reduced invasiveness of reoperations in the nailing group underline the potential advantages of this technique in terms of postoperative management and patient morbidity.

Overall, intramedullary nailing demonstrated faster surgical times, reduced hospital stays, and lower complication rates while maintaining functional outcomes comparable to plate fixation. These findings support the role of nailing as an effective and minimally invasive alternative for selected cases of three-part proximal humerus fractures. Both techniques provided satisfactory radiological and functional recovery. The higher total number of complications reflects the comprehensive inclusion of all adverse events, including minor radiographic findings that did not require reoperation.

## 4. Discussion

Proximal humerus fractures remain a challenging entity in orthopedic trauma, particularly in elderly patients with osteoporotic bone. The management of these injuries continues to be debated, as no single surgical technique has proven universally superior. The results of the present study contribute to this ongoing discussion by directly comparing two widely adopted minimally invasive fixation strategies (plate fixation and intramedullary nailing) applied in two distinct orthopedic centers following consistent inclusion and exclusion criteria.

Our findings confirm that both techniques can provide satisfactory functional and radiological outcomes in the treatment of three-part proximal humerus fractures. However, clear procedural and clinical differences emerged. Intramedullary nailing demonstrated significantly shorter operative and hospitalization times, as well as a reduced rate of postoperative complications compared to plate fixation. These results align with previous reports emphasizing the efficiency of intramedullary fixation in terms of surgical duration, intraoperative blood loss, and soft-tissue preservation [32,33]. The average reduction in surgical time by 55.7% and of hospital stay by 31.5% represents not only a clinical advantage for the patient but also an economic benefit for healthcare systems.

The faster postoperative recovery observed in the nailing group may be explained by the less invasive nature of the technique. The transdeltoid approach and limited soft-tissue dissection allow for early mobilization and reduced pain levels, as confirmed by the significantly lower VAS and higher SST scores at one month post-surgery. Early functional recovery is particularly relevant in elderly patients, where prolonged immobilization is associated with joint stiffness, muscle atrophy, and loss of independence. The faster rehabilitation trajectory following nailing therefore has both clinical and socio-economic implications.

In contrast, plate fixation, although providing excellent visualization and control of the reduction, inevitably requires more extensive dissection and periosteal stripping. This may compromise the vascular supply to the humeral head, predisposing it to avascular necrosis, which was notably more frequent in the plate group of our study. Furthermore, the higher rigidity of the plate–screw construct may lead to stress concentration at the bone–implant interface, explaining the increased incidence of mechanical complications such as loss of fixation and delayed union.

From a biomechanical perspective, the intramedullary nail acts as a load-sharing device positioned along the mechanical axis of the humerus, thereby minimizing bending moments and providing superior resistance to torsional stress [34]. This advantage, combined with reduced soft-tissue trauma, may explain the lower overall complication rate observed in the nail group (30.3% vs. 48.5%) [35]. However, this technique is not free from risks. Incorrect entry point placement may result in iatrogenic damage to the rotator cuff, the supraspinatus tendon, or the greater tuberosity. In addition, inadequate reduction before fixation or suboptimal screw positioning can lead to malalignment, malunion, or screw cut-out [36]. These potential pitfalls underline the necessity of adequate surgical expertise and familiarity with the instrumentation.

Interestingly, although the long-term functional outcomes were similar between the two groups, the early postoperative phase clearly favored nailing. This suggests that, when performed correctly, intramedullary fixation not only shortens operative time but also accelerates the patient’s return to daily activities. The comparable long-term outcomes confirm that both techniques, when properly indicated, provide stable fixation and satisfactory shoulder function, in agreement with the conclusions of Konrad et al. [33] and Domingo et al. [25].

The analysis of complications in relation to Hertel’s fracture classification also revealed a trend toward higher complication rates in more complex fracture types (types 9–11). This observation supports the notion that fracture morphology remains a strong determinant of prognosis, regardless of the fixation method used. In these cases, the surgeon’s ability to achieve and maintain stable reduction is often more relevant than the choice of implant.

The Kaplan–Meier survival analysis further supports these findings, showing that both fixation methods achieved comparable complication-free and reoperation-free survival throughout the follow-up period. The absence of divergence between curves suggests that, despite the higher early complication rate observed in the plate group, the overall long-term safety of the two techniques remains equivalent when appropriate surgical indications and technical accuracy are ensured.

The reoperation rate in our study was lower for nailing (6.1%) compared to plating (9.1%). Moreover, the complexity of secondary procedures differed substantially. Revisions after plate fixation often required conversion to prosthetic replacement due to necrosis or nonunion, whereas those following nailing were limited to minor screw adjustments. These findings reinforce the advantages of nailing in terms of reduced surgical morbidity and easier postoperative management.

Although this study did not include a formal cost analysis, the shorter operative time and reduced hospital stay observed in the intramedullary nailing group may translate into lower healthcare resource utilization and earlier recovery of functional autonomy. These aspects warrant further investigation in prospective cost-effectiveness studies

Despite these favorable results, several limitations must be acknowledged. The retrospective design introduces potential selection bias, and the relatively small sample size limits the statistical power of subgroup analyses. Furthermore, the absence of randomization and the institutional differences in surgical protocols may have influenced some outcomes. Nevertheless, the multicentric design and the inclusion of standardized evaluation parameters lend robustness to the findings.

Future research should focus on prospective randomized studies comparing contemporary plating systems and new-generation nails, possibly incorporating patient-reported outcome measures (PROMs) and cost-effectiveness analyses. Additionally, biomechanical studies investigating the impact of screw trajectory and nail geometry on construct stability could help refine surgical techniques and reduce complication rates.

Our data indicate that intramedullary nailing provides a minimally invasive and efficient alternative to plating for three-part proximal humerus fractures. It achieves comparable functional outcomes while reducing operative time, hospitalization duration, and overall complication rates. Nevertheless, surgical expertise and proper patient selection remain critical to ensure optimal results [37].

The findings are consistent with large meta-analyses and randomized controlled trials comparing intramedullary nailing and locking plate fixation, such as those by Shi et al. [35] and Hohmann et al. [11], which similarly reported faster early recovery and equivalent long-term function. Conversely, other studies have found no clear superiority of either method, emphasizing the need for individualized surgical decision-making. The present results thus align with the broader body of evidence supporting comparable efficacy when technical execution and indication are appropriate.

### Limitations and Strengths of the Study

The present study is not without limitations. First, its retrospective design introduces the inherent risk of selection bias and limits the ability to establish causal relationships between surgical technique and outcomes. Randomization was not performed, and treatment allocation was based on institutional preference, which may have influenced results despite the comparable baseline characteristics between the two groups. The sample size, although adequate for preliminary statistical analysis, remains relatively small for definitive conclusions and may have limited the power to detect subtle differences in functional outcomes or complication subtypes.

Assessors were not fully blinded to the surgical technique and in the evaluation of pain and functional scores, which may have introduced a minor subjective bias in functional scoring.

Because each treatment was performed in a distinct center with different institutional preferences, potential cluster effects related to the surgical team or rehabilitation setting cannot be excluded. Although both institutions followed similar perioperative and rehabilitation protocols, causal inference should be interpreted with caution.

Another limitation lies in the absence of a standardized radiographic evaluation for parameters such as head–shaft angle restoration or screw positioning, which could have provided additional biomechanical insights. Moreover, patient-reported outcome measures (PROMs) beyond the DASH and SST scores were not included, which could have enriched the assessment of quality of life and subjective satisfaction. The relatively short follow-up period, though sufficient to assess fracture healing and early complications, may not fully capture long-term sequelae such as osteoarthritis or late implant failure.

Despite these constraints, the study presents several notable strengths. It is one of the few investigations directly comparing two minimally invasive fixation techniques for three-part proximal humerus fractures under consistent inclusion and exclusion criteria. The homogeneity of implants used in each group minimizes variability related to device design. Furthermore, all procedures were performed by experienced upper limb trauma surgeons, ensuring high technical consistency. The combination of functional, clinical, and radiographic evaluations at multiple time points provides a comprehensive overview of patient recovery and treatment efficacy. Finally, the multicentric design, involving two geographically distinct hospitals, enhances the generalizability of the findings and reflects real-world clinical practice.

Future prospective, randomized, and multicentric studies with larger patient populations and standardized rehabilitation protocols are warranted to confirm these results and further refine the decision-making process for optimal surgical management of proximal humerus fractures.

## 5. Conclusions

The management of proximal humerus fractures remains a complex challenge, especially in elderly patients with osteoporotic bone. This two-center comparative study demonstrates that intramedullary nailing provides significant advantages over plate fixation in the treatment of three-part proximal humerus fractures. Specifically, it offers reduced operative and hospitalization times, a lower incidence of postoperative complications, and a faster return to functional activity. Despite these benefits, long-term outcomes in terms of shoulder function and radiographic healing were comparable between the two techniques.

While intramedullary nailing appears to offer practical advantages in selected cases, its adoption as a first-line treatment should be tempered by patient-specific factors and surgeon expertise. Conversely, plate fixation remains valuable for cases requiring precise anatomical reduction or management of more complex fracture patterns. Ultimately, the choice between these two techniques should be based on a thorough preoperative assessment that considers fracture morphology, patient-specific factors such as bone quality and functional demand, and the surgeon’s expertise with the chosen device.

Future randomized prospective trials with larger patient populations and longer follow-up durations are needed to further validate these findings and to better define evidence-based indications for each fixation method. Studies integrating patient-reported outcomes and cost–benefit analyses could also enhance our understanding of the true clinical and economic impact of these procedures.

## Figures and Tables

**Table 1:** Baseline demographic and perioperative characteristics of patients treated with plate fixation and intramedullary nailing for three-part proximal humerus fractures.

Variable	Plate Fixation (*n* = 33)	Intramedullary Nailing (*n* = 33)	*p*-Value (95% CI)
Age, years (mean ± SD)	67.9 ± 4.3	67.6 ± 4.1	0.78
Sex (F/M)	24/9	25/8	0.79
Dominant side affected, *n* (%)	17 (51.5%)	16 (48.5%)	0.82
ASA ≥ III, *n* (%)	7 (21.2%)	6 (18.2%)	0.74
Diabetes mellitus, *n* (%)	5 (15.2%)	4 (12.1%)	0.72
Current smoker, *n* (%)	6 (18.2%)	7 (21.2%)	0.76
Chronic steroid use, *n* (%)	3 (9.1%)	2 (6.1%)	0.64
Injury mechanism (low-energy fall), *n* (%)	29 (87.9%)	28 (84.8%)	0.68
Preoperative waiting time, days (mean ± SD)	2.3 ± 0.9	2.1 ± 1.0	0.44
Operative time, min (mean ± SD)	62.1 ± 9.7	27.3 ± 5.9	<0.001
Hospital stay, days (mean ± SD)	9.2 ± 2.5	6.3 ± 2.1	<0.001
Healing time, days (mean ± SD)	88.2 ± 18.3	78.4 ± 14.7	0.04

“Hospitalization period” refers to the total number of days of in-hospital stay. “Healing time” corresponds to the interval between surgery and radiographic evidence of cortical and trabecular bony union in two orthogonal views. No significant differences were observed between groups for age, sex distribution, comorbidities, or injury mechanism. Operative time and hospital stay were significantly shorter in the nailing group.

**Table 2:** Comparison of postoperative functional and radiological outcomes between plate fixation and intramedullary nailing.

Outcome	Plate Fixation	Intramedullary Nailing	*p*-Value (95% CI)
SST score (0–12), 1 month	6.8 ± 1.4	8.2 ± 1.2	0.003
SST score (0–12), 3 months	8.7 ± 1.0	9.1 ± 0.9	0.21
SST score (0–12), 6 months	9.6 ± 1.0	9.8 ± 0.8	0.42
SST score (0–12), 12 months	10.1 ± 0.8	10.3 ± 0.7	0.38
DASH, 1 month	48.5 ± 8.1	42.7 ± 7.5	0.01
DASH, 12 months	18.3 ± 5.2	17.1 ± 5.4	0.42
VAS pain (0–10), 1 month	3.8 ± 1.0	2.9 ± 0.8	0.002
VAS pain (0–10), 12 months	1.2 ± 0.6	1.0 ± 0.5	0.19
Barthel Index, 1 month	78.2 ± 9.5	84.1 ± 8.7	0.01
Barthel Index, 12 months	93.8 ± 5.2	94.1 ± 4.8	0.79
Forward elevation (°), final	135 ± 21	139 ± 18	0.48
Abduction (°), final	128 ± 22	132 ± 19	0.52
Complications, *n* (%)	16 (48.5%)	10 (30.3%)	0.09
Reoperations, *n* (%)	3 (9.1%)	2 (6.1%)	0.64

Intramedullary nailing showed faster early recovery in SST, VAS, and Barthel scores, while long-term functional outcomes and range of motion were comparable between groups.

**Table 3:** Distribution of postoperative complications in patients treated with plate fixation and intramedullary nailing for three-part proximal humerus fractures.

	Plate	Nail
Malunion	4	4
Necrosis	4	/
Loss of reduction	3	1
Intraoperative fracture	/	1
Cuff lesion	/	1
Screw cut-out	1	2
Delayed union	3	1
Nonunion	1	/

Complications are categorized by type and surgical technique. Values represent the number of affected patients in each group. Overall complication rates were 48.5% in the plate group and 30.3% in the nail group. Avascular necrosis and loss of reduction occurred more frequently after plate fixation, whereas screw cut-out and cuff lesions were observed exclusively in the nailing group. No deep infections were recorded.

## Data Availability

The data presented in this study are available on request from the corresponding author.

## References

[1] Biberthaler P., Wijdicks J.P. (2015). Fractures of the Proximal Humerus.

[2] Kim S.H., Szabo R.M., Marder R.A. (2012). Epidemiology of humerus fractures in the United States: Nationwide emergency department sample, 2008. Arthritis Care Res..

[3] Court-Brown C.M., Cattermole H., McQueen M.M. (2002). Impacted valgus fractures (B1.1) of the proximal humerus: The results of non-operative treatment. J. Bone Jt. Surg. Br..

[4] Koob S., Plöger M.M., Bornemann R., Lehmann R.P., Alex D., Placzek R. (2022). Intramedullary Nailing Versus Compound Plate Osteosynthesis in Pathologic Diaphyseal Humerus Fractures: A Retrospective Cohort Study. Am. J. Clin. Oncol..

[5] Martinez-Catalan N., Boileau P. (2023). The Role of Intramedullary Nailing for Proximal Humerus Fractures: What Works and What Does Not. Curr. Rev. Musculoskelet. Med..

[6] Pandey R., Raval P., Manibanakar N., Nanjayan S., McDonald C., Singh H. (2023). Proximal humerus fractures: A review of current practice. J. Clin. Orthop. Trauma.

[7] Wu K., Lin T., Lee C.H. (2023). Intramedullary nailing versus cemented plate for treating metastatic pathological fracture of the proximal humerus: A comparison study and literature review. J. Orthop. Traumatol..

[8] Scaravilli G., Mercurio J., Grazioli A., Gioia C., D’Arienzo A., Toro G., Galvano N., Monteleone G., Battaglini G., Di Santo F. (2022). Preliminary report in treatment of proximal humeral fracture with closed reduction and DOS external fixation System: A multicentric study. Acta Biomed..

[9] Rudran B., Little C., Duff A., Poon H., Tang Q. (2022). Proximal humerus fractures: Anatomy, diagnosis and management. Br. J. Hosp. Med..

[10] Schultz B.J., Lowe D.T., Egol K.A., Zuckerman J.D. (2021). Shoulder Hemiarthroplasty for Proximal Humerus Fracture. J. Orthop. Trauma.

[11] Hohmann E., Keough N., Glatt V., Tetsworth K. (2023). Surgical treatment of proximal humerus fractures: A systematic review and meta-analysis. Eur. J. Orthop. Surg. Traumatol..

[12] Crosby L.A., Neviaser R.J., Warner J.J.P., Iannotti J.P. (2015). Proximal humerus fractures. Evaluation and Management of Shoulder Disorders.

[13] Iannotti J.P., Ramsey M.L., Williams J.G.R., Warner J.J. (2004). Nonprosthetic management of proximal humeral fractures. Instr. Course Lect..

[14] Neer C.S. (2002). Four-segment classification of proximal humeral fractures: Purpose and reliable use. J. Shoulder Elb. Surg..

[15] Lill H., Josten C. (2000). Proximal and distal humerus fractures in advanced age. Orthopade.

[16] Szyszkowitz R., Schippinger G. (1999). Fractures of the proximal humerus. Unfallchirurg.

[17] Resch H. (2003). Fractures of the humeral head. Unfallchirurg.

[18] Neer C.S. (1970). Displaced proximal humeral fractures. I. Classification and evaluation. J. Bone Jt. Surg..

[19] Meinberg E.G., Agel J., Roberts C.S., Karam M.D., Kellam J.F. (2018). Fracture and dislocation classification compendium—2018. J. Orthop. Trauma.

[20] Hertel R., Hempfing A., Stiehler M., Leunig M. (2004). Predictors of humeral head ischemia after intracapsular fracture of the proximal humerus. J. Shoulder Elb. Surg..

[21] Kettler M., Biberthaler P., Braunstein V., Zeiler C., Kroetz M., Mutschler W. (2006). Treatment of proximal humeral fractures with the PHILOS angular stable plate. Presentation of 225 cases of dislocated fractures. Unfallchirurg.

[22] Tepass A., Blumenstock G., Weise K., Rolauffs B., Bahrs C. (2013). Current strategies for the treatment of proximal humeral fractures: An analysis of a survey carried out at 348 hospitals in Germany, Austria, and Switzerland. J. Shoulder Elb. Surg..

[23] Martetschläger F., Siebenlist S., Weier M., Sandmann G., Ahrens P., Braun K., Elser F., Stöckle U., Freude T. (2012). Plating of proximal humeral fractures. Orthopedics.

[24] Jabran A., Peach C., Ren L. (2018). Biomechanical analysis of plate systems for proximal humerus fractures: A systematic literature review. Biomed. Eng. Online.

[25] Trepat A.D., Popescu D., Fernández-Valencia J.A., Cuñé J., Rios M., Prat S. (2012). Comparative study between locking plates versus proximal humeral nail for the treatment of 2-part proximal humeral fractures. Eur. J. Orthop. Surg. Traumatol..

[26] Handoll H.H., Elliott J., Thillemann T.M., Aluko P., Brorson S. (2022). Interventions for treating proximal humeral fractures in adults. Cochrane Database Syst. Rev..

[27] Maniscalco P., Ciatti C., Gattoni S., Quattrini F., Puma Pagliarello C., Patane’ A.C., Capelli P., Banchini F., Rivera F., Sanna F. (2021). Proximal humerus fractures in COVID-19 lockdown: The experience of three orthopedics and traumatology departments in the first ten weeks of the Italian epidemic. Acta Biomed..

[28] Maniscalco P., Quattrini F., Ciatti C., Gattoni S., Puma Pagliarello C., Burgio V., Di Stefano G., Cauteruccio M., Giovanelli M., Magro A. (2020). The Italian COVID-19 Phase 2 in Piacenza: Results of the first semester of 2020 and future prospective of new orthopedics surgical procedures. Acta Biomed..

[29] Gummesson C., Atroshi I., Ekdahl C. (2003). The disabilities of the arm, shoulder and hand (DASH) outcome questionnaire: Longitudinal construct validity and measuring self-rated health change after surgery. BMC Musculoskelet. Disord..

[30] Collin C., Wade D.T., Davies S., Horne V. (1988). The Barthel ADL Index: A reliability study. Int. Disabil. Stud..

[31] Godfrey J., Hamman R., Lowenstein S., Briggs K., Kocher M. (2007). Reliability, validity, and responsiveness of the simple shoulder test: Psychometric properties by age and injury type. J. Shoulder Elb. Surg..

[32] Shi X., Liu H., Xing R., Mei W., Zhang L., Ding L., Huang Z., Wang P. (2019). Effect of intramedullary nail and locking plate in the treatment of proximal humerus fracture: An update systematic review and meta-analysis. J. Orthop. Surg. Res..

[33] Konrad G., Audige L., Lambert S., Hertel R., Su N.P. (2012). Similar Outcomes for Nail versus Plate Fixation of Three-part Proximal Humeral Fractures. Clin. Orthop. Relat. Res..

[34] Clavert P., Hatzidakis A.M., Boileau P. (2016). Anatomical and biomechanical evaluation of an intramedullary nail for fractures of proximal humerus fractures based on tuberosity fixation. Clin. Biomech..

[35] Aguado H.J., Mingo J., Torres M., Alvarez-Ramos A., Martín-Ferrero M.A. (2016). Minimally invasive polyaxial locking plate osteosynthesis for 3–4 part proximal humeral fractures: Our institutional experience. Injury.

[36] Euler S.A., Hengg C., Kolp D., Wambacher M., Kralinger F. (2014). Lack of fifth anchoring point and violation of the insertion of the rotator cuff during antegrade humeral nailing: Pitfalls in straight antegrade humeral nailing. Bone Jt. J..

[37] Quattrini F., Ciatti C., Gattoni S., Burgio V., Puma Pagliarello C., Rivera F., Maniscalco P. (2022). DIPHOS^®^ nail for proximal humeral fractures: Our experience with more than 190 procedures and surgical tips. Acta Biomed..

